# The Attitudes and Perceptions of Israeli Psychiatrists Toward Telepsychiatry and Their Behavioral Intention to Use Telepsychiatry

**DOI:** 10.3389/fpsyt.2022.829965

**Published:** 2022-03-21

**Authors:** Hanoch Kaphzan, Margaret Sarfati Noiman, Maya Negev

**Affiliations:** ^1^Department of Neurobiology, Faculty of Natural Sciences, University of Haifa, Haifa, Israel; ^2^Faculty of Social Welfare and Health Sciences, University of Haifa, Haifa, Israel; ^3^Division of Health Systems Policy and Administration, Faculty of Social Welfare and Health Sciences, University of Haifa, Haifa, Israel

**Keywords:** telepsychiatry, telemedicine, unified theory of acceptance and the use of technology, mental health services, barriers and facilitative factors

## Abstract

**Background:**

Although telemedicine care has grown in recent years, telepsychiatry is growing at a slower pace than expected, because service providers often hamper the assimilation and expansion of telepsychiatry due their attitudes and perceptions. The unified theory of acceptance and use of technology (UTAUT) is a model that was developed to assess the factors influencing the assimilation of a new technology. We used the UTAUT model to examine the associations between the attitudes and perceptions of psychiatrists in Israel toward telepsychiatry and their intention to use it.

**Methods:**

An online, close-ended questionnaire based on a modified UTAUT model was distributed among psychiatrists in Israel. Seventy-six questionnaires were completed and statistically analyzed.

**Results:**

The behavioral intention of Israeli psychiatrists to use telepsychiatry was relatively low, despite their perceptions of themselves as capable of high performance with low effort. Nonetheless, they were interested in using telepsychiatry voluntarily. Experience in telepsychiatry, and to a lesser extent, facilitating conditions, were found to be positively correlated with the intention to use telepsychiatry. Psychiatrists have a positive attitude toward treating patients by telepsychiatry and perceive its risk as moderate.

**Discussion:**

Despite high performance expectancy, low effort expectancy, low perceived risk, largely positive attitudes, high voluntariness, and the expectancy for facilitating conditions, the intention to use telepsychiatry was rather low. This result is explained by the low level of experience, which plays a pivotal role. We recommend promoting the facilitating conditions that affect the continued use of telepsychiatry when initiating its implementation, and conclude that it is critical to create a sense of success during the initial stages of experience.

## Introduction

In the Western world, increases in both life expectancy and chronic morbidity have greatly enhanced the demand for medical services, resulting in an overburdened health system. One of the main difficulties that public mental health services are facing is the discrepancy between supply and demand, as mental disorders are among the most prevalent illnesses in Western civilization (1), while there is a global shortage of mental health professionals in general, and of psychiatrists, in particular. The global median number of psychiatrists is ~1:100,000 people, making them a rare resource in mental health systems around the world (2). In an attempt to mitigate the shortage in human medical resources, to increase access to mental health services, and to reduce psychiatrists' burnout without compromising quality and satisfaction, there is a recommendation to use telemedicine in mental health (3, 4). Telemedicine is defined as “the delivery of health care and the exchange of health-care information across distances” (5). Previous research supports the claim that telehealth interventions can effectively shorten waiting lists and improve the coordination of specialist services (6).

Within telemedicine, telepsychiatry is defined as the use of communication technology to provide or support psychiatric services across distances. It usually refers to video-calls, which enable interactive, live, and colorful two-way communication (7). Randomized controlled trials comparing video-based interventions with face-to-face (FTF) interventions have shown that in most of the disorders examined—depression (8) anxiety (9), post-trauma (10), eating disorders (11), substance abuse (12), and suicide prevention (13)—the outcomes are comparable. Moreover, other studies have suggested that mental health care provided *via* video calls is equivalent to FTF interventions for creating and maintaining a solid rapport, and a satisfying therapeutic relationship between physicians and patients (14, 15). In general, both providers and patients were satisfied with telepsychiatry and found it acceptable, but patients reported higher satisfaction than did service providers. In addition, telepsychiatry has some economic benefits, as it reduces the direct and indirect costs of health care and increases the quality of life of patients in adjusted years when compared to FTF (16).

Although telemedicine care has increased significantly in recent years, telepsychiatry assimilation has been slower than expected, because the service providers, not the patients, have hampered its assimilation and expansion (17). The most frequently identified barriers for its acceptance were the licensing regulations, risks to patient confidentiality, concerns regarding reimbursement of expenses, patient safety, and interoperability (16, 18, 19). Clinicians' perspectives served as gatekeepers for the implementation and sustainability of telepsychiatry services (17), and despite the multiple benefits of telepsychiatry, studies have shown that various barriers prevent its assimilation and optimal use, and it is critical that psychiatrists support telepsychiatry (20). If psychiatrists are not prepared to use it at the clinical level, it will not be accessible for the patient to use (21).

In Israel, the therapeutic mental health care system is based in the community and supplied by the health maintenance organizations (HMOs). Accessibility to health services varies between regions, being usually lower in the periphery (22), and there is a severe shortage of psychiatrists (23). Hence, telepsychiatry has the potential to alleviate this shortage by enhancing the efficiency of mental health services. Therefore, the need to assess barriers and incentives for the use of telepsychiatry in Israel has emerged.

One of the most widely used models for investigating the acceptance of new technologies is the unified theory of acceptance and use of technology (UTAUT) model, aimed at understanding the concept of “actual use” and its estimation. The term “behavioral intention to use” was formulated to predict the behavior of using a technology (24).

The present study seeks to examine various aspects related to psychiatrists attitudes toward telepsychiatry. In particular, we aim to examine the extent to which psychiatrists in Israel show a behavioral intention to use telepsychiatry, and to identify the factors that may constitute an obstacle, or alternatively, encourage psychiatrists to use telepsychiatry. Assessment of the intent to use telepsychiatry was based upon variables derived from the UTAUT model using a validated questionnaire. This study, which was executed before the use of telemedicine services became more frequent in Israel during COVID-19, may provide a baseline for further studying the use of telepsychiatry. In addition, it can enable a better understanding of the barriers and incentives toward telepsychiatry assimilation amongst psychiatrists. This comprehension can assist in designing an effective policy to make psychiatric services more accessible.

**Figure 1:**
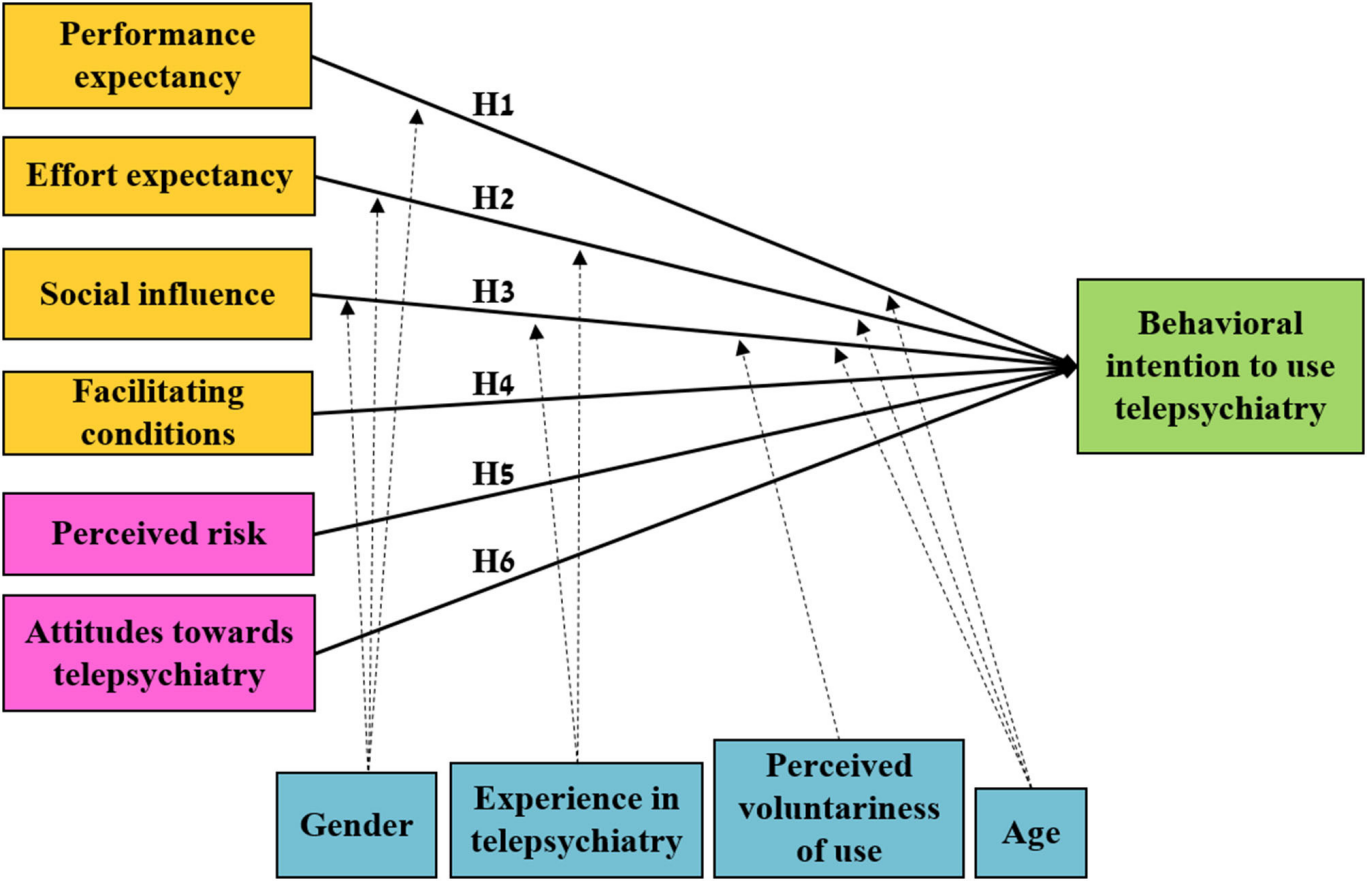
An illustration of the modified UTAUT model used in the study. The model is similar to the UTAUT model, except for the addition of the perceived risk and attitudes toward telepsychiatry variables. Orange and pink—independent variables, blue—confounding variables (covariables), green—dependent variable.

Based on the UTAUT model (Figure 1) (24), we hypothesize that:

H1: The effect of performance expectancy on behavioral intention will be correlated with gender and age. We predict that the correlation will be stronger for men, particularly younger men.
H2: The effect of effort expectancy on behavioral intention will be correlated with gender, age, and experience. We predict that the correlation will be stronger for women, particularly younger women, and particularly in the early stages of experience.
H3: The effect of social influence on behavioral intention will be correlated with gender, age, voluntariness, and experience. We predict that the correlation will be stronger for women, particularly older women, particularly in mandatory settings in the early stages of experience.
H4: Facilitating conditions will not be significantly correlated with behavioral intention.

In addition, based on previous findings that attitudes toward technology (25) and perceived risk of practicing electronic health (26) are correlated to behavioral intention to practice health technology (Figure 1), we hypothesis that:

H5: Perceived risk will be negatively correlated to behavioral intention.
H6: Attitudes toward telepsychiatry will be positively correlated with behavioral intention.

## Materials and Methods

We used a modified version of the UTAUT model (Figure 1) (24). Since telepsychiatry is not yet commonly used in Israel, we defined “behavioral intention to use telepsychiatry” as the dependent variable in our study, and tested its association with four independent variables. Three of these variables have been found to be associated with the intention to use: Performance expectancy, Effort expectancy, and Social influence. The fourth variable, Facilitating conditions, is associated with actual use (24). All variables are defined in Supplementary Table 1. In addition, the UTAUT model contains four confounding variables (i.e., covariables): gender, age, experience, and perceived voluntariness of use. Perceived voluntariness of use reflects the interest in using telepsychiatry voluntarily, and not only when mandatory. The framework of this model frequently requires modification for its application in complex health care settings (19).

To examine the attitudes of psychiatrists toward confidentiality and privacy when using telepsychiatry, we defined the variable, perceived risk, as the potential for loss in the pursuit of a desired outcome of using an e-service (27). The UTAUT model also incorporates attitudes toward telemedicine and the use of technology in general, but to estimate these two elements, we used two additional questionnaires; the attitude toward telemedicine in psychiatry and psychotherapy (ATIPP) questionnaire, and the attitude toward using technology questionnaire. The ATIPP questionnaire examines the attitudes of psychiatrists toward specific aspects of telemedicine in mental health (25), while the attitude toward using technology questionnaire estimates an individual's overall affective reaction to using a system (24). Altogether, according to the UTAUT model, all of the aforementioned variables should be associated with the outcome of behavioral intention, which refers to the individual's motivation regarding the performance of a given behavior (28).

The questionnaire used in this study was based on previous UTAUT model studies and was distributed online using Qualtrics software. The study used a convenience sampling of psychiatrists who practice psychiatry in Israel. The questionnaire was distributed *via* lists of email addresses of all registered psychiatrists in the Israeli Medical Association (IMA). The survey data were collected from November 2019 to April 2020, mostly before the COVID-19 pandemic. The study was ethically approved by the University of Haifa Ethics Committee.

### Participants

The participants were Israeli board-certified psychiatrists who work in Israel's public and private health systems, and psychiatry residents who work in Israel's public health system. The survey was based on responses to an online questionnaire. The questionnaire was anonymous and participants stated that they were psychiatrists and agreed to participate in the survey.

The survey link was sent by email *via* the Israeli Psychiatric Association (IPA), which is the official union of psychiatrists within the IMA. The link was sent to all member psychiatrists including managers of psychiatric hospitals, psychiatric departments in general hospitals, mental health centers and psychiatric clinics. Subsequently, the managers of these facilities were asked several times to encourage and distribute the survey among the psychiatrists in their facilities.

Out of 100 respondents who agreed to participate in the online survey, 76 completed the survey. The remaining 24 respondents began the survey but did not complete it and their responses were excluded.

### Measures and Questionnaires

The survey comprised demographic variables (age, gender, religion, native language, years of experience in psychiatry, geographical area of work, job title, and place of work). Each of the *independent variables* of UTAUT—Performance expectancy, Effort expectancy, Social influence, and Facilitating conditions—were tested using four questions, and the dependent variable, behavioral intention, was tested using three questions. The variables, experience, and perceived voluntariness of use were tested using one question each (24). Attitude toward using technology, was tested using the ATIPP questionnaire, comprising eight questions; Cronbach's alpha for the physicians' version of the questionnaire used in this study was 0.827 (25). Perceived risk—a question taken from a study about physicians' perceptions of trust in the system and risk-related factors, was divided into two questions in the present study (26). In the demographic questionnaire, psychiatrists could choose more than one option when answering the question about job title and place of work.

All variables were measured on a 5-point Likert scale ranging from strongly disagree to strongly agree, except for experience, which was measured on a scale of 1–3 (not at all, little and much experience). For statistical calculations, the scores for this variable were divided into two groups: without experience (1) and with experience (2-3). For variables with more than one question, the mean value was used for statistical analyses.

Since telemedicine has not been used often in Israel, some of the questions in the UTAUT model were adjusted to future tense. The questionnaire was delivered in Hebrew. To confirm the quality of translation, the questions were translated into Hebrew and then back-translated to English by independent qualified personnel. Following the back translation, the two English versions were compared.

The English versions of the UTAUT and ATIPP questionnaires are in the Supplementary Materials.

### Data Analysis

The Qualtrics online survey responses were exported to SPSS. Descriptive statistics were calculated for the demographic variables (mean, standard deviation, frequency, and percentages). Three hypotheses, H1, H2, and H3, were analyzed using linear regressions (unstandardized coefficients B, unstandardized coefficients standard error, and adjusted *R*^2^). The other hypotheses, H4, H5, and H6, were tested using Pearson correlations. Significance was determined at *P* < 0.05. The rationale for presenting the separate regressions for each hypothesis is the small *N*.

## Results

One hundred psychiatrists initially responded to the survey, of whom 76 psychiatrists completed the survey and participated in the study. This represents ~10% of the practicing psychiatrists in Israel. The data collected included demographic data (Table 1) and data related to their professional occupation as psychiatrists (Table 2). The study sample had similar participation of men and women (male = 39, female = 37) (Table 1). The age of the participants ranged from 28 to 77. Most participants were of Jewish origin (88%) and most participants worked in Tel Aviv and the central region of Israel (31.6%), a fair representation of the population distribution in the country which is 40% in Tel Aviv and the Central District, 13% in the Jerusalem District, 29% in Haifa and the Northern District, and 15% in the South and Shfela (29) (Table 2).

**Table 1:** Demographic data of the survey participants.

**Characteristic**	***n***	**%**
**Gender**		
Female	37	51.3
Male	39	48.7
**Age**		
28–37	11	14.5
38–47	30	39.5
48–57	18	23.7
58–67	12	15.8
68–77	5	6.6
**Religion**		
Jewish	67	88.2
Christian	1	1.3
Muslim	3	3.9
Druze	0	0
Other	5	6.6
**Native language**		
Arabic	5	6.6
Hebrew	49	64.5
Russian	14	18.4
English	3	3.9
Other	5	6.6
Total	76	100

**Table 2:** Profession-related data of the survey participants (*n* = 74, as two participants did not provide demographic data).

**Parameter**	***N***			**%**
**Years of experience in psychiatry**					
Resident in psychiatry	9			11.8
Up to 5	9			11.8
6–10	12			15.8
11–15	11			14.5
16–20	9			11.8
21–25	8			10.5
26+	16			21.1
**Geographical area of work**					
Northern District	9			11.8
Haifa District	13			17.1
The Triangle and Sharon Region	11			14.5
Tel Aviv and Central District	24			31.6
Jerusalem District	8			10.5
Shfela Region	3			3.9
Southern District	6			7.9
**Job title**	**Main workplace**		**Secondary workplace**	
	***n***	**%**	***N***	**%**
Psychiatry resident	23	30.3	7	9.2
Psychiatry consultant	25	32.9	10	13.2
Service Deputy/Manager	10	13.2	3	3.9
Clinic Deputy/Manager	9	11.8	1	1.3
Department Deputy/Manager	14	18.4	0	0
Division Deputy/Manager	6	7.9	0	0
**Place of work**	**Main workplace**		**Secondary workplace**	
	***n***	**%**	***N***	**%**
Department of a general hospital	6	7.9	1	1.3
Department of a psychiatric hospital	25	32.9	1	1.3
Mental health clinic in a general hospital	10	13.2	1	1.3
Mental health clinic in a psychiatric hospital	8	10.5	1	1.3
Community mental health clinic	11	14.5	2	2.6
Psychiatrist at an HMO health clinic	12	15.8	6	7.9
Psychiatrists within the social services	0	0	1	1.3
Hostel	0	0	4	5.3
Private clinic	16	21.1	29	38.2
Other	3	3.9	2	2.6
**Experience in telemedicine**	***N***	**%**		
Without experience	42	55.3		
Little experience	27	35.5		
Much experience	7	9.2		

The distribution of years of experience was quite homogenous, with 21% of participants having over 26 years of experience (Table 2). The position levels—resident, consultant, or managerial position—were also distributed almost equally between these three levels. Psychiatrists could choose more than one option if they worked as both consultants and managers. Nearly one third of the participants worked in a department within a mental health institute as their primary workplace, while the others were distributed among other workplaces. Approximately 59% of psychiatrists also worked in a private clinic, either as their primary workplace (main position and time dedication) or their secondary workplace (Table 2).

Since our primary reference framework was the UTAUT model, data are presented in accordance with the UTAUT variables. Cronbach's alpha mostly ranged from acceptable to good (0.668–0.975), confirming a reasonable to high internal consistency within our questionnaire, with the exception of the social influence, which had a value of 0.67 (Table 3). Experience using the technology and voluntariness to use the technology were each based on a single question (Table 3).

**Table 3:** Descriptive statistics from the survey used in the present study.

	**Mean**	**Standard deviation**	**Cronbach's alpha**
**Variables from the UTAUT model**			
**Performance expectancy (mean)**	3.64	0.85	0.858
I would find the system useful in my job	3.95	0.96	
Using the system will enable me to accomplish tasks more quickly	3.63	1.03	
Using the system will increase my productivity	3.57	1.02	
If I use the system, I will increase my chances of getting a raise	3.43	1.05	
**Effort expectancy (mean)**	3.79	0.77	0.888
My interaction with the system will be clear and understandable	3.51	0.90	
It will be easy for me to become skillful at using the system.	3.92	0.87	
I will find the system easy to use	3.76	0.92	
Learning to operate the system will be easy for me	4.00	0.86	
**Social influence (mean)**	3.36	0.62	0.668
People who influence my behavior will think that I should use the system	3.32	0.91	
People who are important to me will think that I should use the system	3.32	0.80	
The senior management of this organization will be helpful in the use of the system	3.34	0.98	
In general, the organization will support the use of the system	3.47	0.82	
**Facilitating conditions (mean)**	3.60	0.63	0.749
I will have the resources necessary to use the system	3.45	0.87	
I will have the knowledge necessary to use the system	3.99	0.73	
The system will be compatible with other systems I use	3.57	0.95	
A specific person (or group) is available for assistance with system difficulties	3.42	0.80	
**Behavioral intention to use the system (mean)**	2.92	1.19	0.975
I intend to use the system in the next 6 months	2.92	1.20	
I predict I will use the system in the next 6 months	3.01	1.22	
I plan to use the system in the next 6 months	2.84	1.24	
**Experience in telemedicine**	2.07	1.32	
**Voluntariness**	3.70	1.16	
**Variables added to the UTAUT model**			
**Perceived risk** (mean)	2.55	0.90	0.867
**Attitude toward telepsychiatry** (mean)	3.79	0.63	0.797

Overall, the data show that although the values of the independent variables (Effort expectancy, Performance expectancy, Social influence and Facilitating conditions) were rather high (3.4–3.8), including Perceived voluntariness of use (3.70), the dependent variable of the model (Behavioral intention) was quite low (2.9) (Table 3). The only independent variable that was also low was personal experience in telepsychiatry (Experience = 2.07) (Table 3). This variable comprised 55.3% psychiatrists without experience, 35.5% with little experience, and only 9.2% with much experience. Despite the average low experience, Israeli psychiatrists perceive themselves as capable of high performance (3.64) and believe that using telepsychiatry will require a low effort (3.70) (Table 3). Furthermore, they perceive the facilitating conditions as being quite good (3.60) (Table 3).

We note that the variables added to the UTAUT model in the present study, perceived risk and attitudes, indicated a largely positive attitude toward telepsychiatry (3.79) with a low perceived risk (2.55) (Table 3).

### Behavioral Intention to Use Telepsychiatry Is Correlated With Performance Expectancy, Age, and Gender (H1)

The linear regression between the dependent variable, behavioral intention to use telepsychiatry, and the independent variables, performance expectancy, age, and gender, showed that behavioral intention to use telepsychiatry was strongly correlated with performance expectancy and gender [*F*_(3,72)_ = 9.933, *P* <0.01, adjusted *R*^2^ = 0.263] (Table 4). The correlation was stronger for men. Interestingly, there was no correlation with age.

**Table 4:** Linear regression of behavioral intention to use telepsychiatry on performance expectancy, age, and gender.

**Independent variable**	**Unstandardized coefficients**	**Unstandardized coefficients**	**Sig**.
	**B**	**Standard error**	
Age	0.212	0.109	0.057
Gender	−0.642	0.240	0.009
Performance expectancy	0.643	0.142	0.001

### Behavioral Intention to Use Telepsychiatry Is Correlated With Effort Expectancy and Experience (H2)

Similarly, linear regression analysis show that behavioral intention to use telepsychiatry, and the independent variables, effort expectancy, experience, age, and gender, showed that behavioral intention to use telepsychiatry was strongly correlated with effort expectancy and experience [*F*_(4,71)_ = 10.83, *P* < 0.01, and adjusted *R*^2^ = 0.344] (Table 5); however, there was no correlation with age or gender.

**Table 5:** Linear regression of behavioral intention to use telepsychiatry on effort expectancy, experience, age, and gender.

**Independent variable**	**Unstandardized coefficients**	**Unstandardized coefficients**	**Sig**.
	***B***	**Standard error**	
Effort expectancy	0.593	0.156	0.001
Experience	0.759	0.237	0.002
Age	0.125	0.103	0.229
Gender	−0.342	0.235	0.151

### Behavioral Intention to Use Telepsychiatry Is Correlated With Experience and Voluntariness (H3)

The linear regression analysis of the dependent variable, behavioral intention to use telepsychiatry, on the independent variables, social influence, experience, age, gender, and voluntariness, showed that behavioral intention to use telepsychiatry was strongly correlated with experience in using telepsychiatry and voluntariness [*F*_(5,70)_ = 8.108, *P* < 0.01, and adjusted *R*^2^ = 0.322] (Table 6). However, there was no correlation with age, gender, or social influence.

**Table 6:** Linear regression of behavioral intention to use telepsychiatry on experience, age, gender, social influence, and perceived voluntariness of use.

**Independent variable**	**Unstandardized coefficients**	**Unstandardized coefficients**	**Sig**.
	***B***	**Standard error**	
Experience	0.848	0.249	0.001
Age	0.107	0.106	0.318
Gender	−0.493	0.236	0.040
Social Influence	0.273	0.199	0.175
Perceived Voluntariness	0.277	0.111	0.015

### The Relationship Between Behavioral Intention to Use Telepsychiatry and Facilitating Conditions (H4)

The Pearson correlation analysis between behavioral intention to use telepsychiatry and facilitating conditions showed a strong to moderate positive relationship [*r*_(76)_ = 0.45, *p* < 0.01] (Table 7). This finding was in contrast to our hypothesis that facilitating conditions will not have a significant effect on behavioral intention.

**Table 7:** Pearson correlation between all study variables.

	**Performance expectancy**	**Effort expectancy**	**Social influence**	**Facilitating conditions**	**Experience**	**Voluntariness**	**Attitude toward telepsychiatry**	**Perceived risk**	**Behavioral intention**
**Performance expectancy**	1	0.563**	0.518**	0.520**	0.151	0.748**	0.746**	−0.614**	0.447**
**Effort expectancy**		1	0.456**	0.586**	0.270*	0.602**	0.675**	−0.498**	0.488**
**Social influence**			1	0.515**	−0.098	0.339**	0.539**	−0.282*	0.211
**Facilitating conditions**				1	0.122	0.593**	0.655**	−0.474**	0.455**
**Experience**					1	0.258*	0.210	−0.280*	0.457**
**Voluntariness**						1	0.756**	−0.696**	0.410**
**Attitude toward telepsychiatry**							1	−0.709**	0.472**
**Perceived risk**								1	−0.375**
**Behavioral intention**									1

*Correlation is significant at P < 0.05 (2-tailed), **correlation is significant at P < 0.01 (2-tailed).

### The Relationship Between Behavioral Intention to Use Telepsychiatry and Perceived Risk (H5)

The Pearson correlation between behavioral intention to use telepsychiatry and perceived risk showed a moderate, negative relationship [*r*_(76)_ = −0.37, *p* < 0.01] (Table 7). This finding is consistent with our hypothesis that perceived risk will be negatively correlated with behavioral intention.

### The Relationship Between Behavioral Intention to Use Telepsychiatry and Psychiatrists' Attitudes Toward Telepsychiatry (H6)

The Pearson correlation analysis between behavioral intention to use telepsychiatry and psychiatrists' attitudes toward telepsychiatry showed a strong to moderate positive relationship [*r*_(76)_ = 0.47, *p* < 0.01] (Table 7). This finding is consistent with our hypothesis that attitudes toward telepsychiatry will have a significant effect on behavioral intention to use telepsychiatry.

### The Relationships Between Behavioral Intention to Use Telepsychiatry and the Independent Variables

The Pearson correlation analyses between the dependent variable, behavioral intention to use telepsychiatry, and the independent variables showed strong to moderate positive relationships with some of them, such as facilitating conditions [*r*_(76)_ = 0.45, *P* < 0.01], attitudes toward telepsychiatry [*r*_(76)_ = 0.47, *P* < 0.01], and a moderate negative relationship with perceived risk [*r*_(76)_ = −0.37, *P* < 0.01].

In each of the models where the independent variable, psychiatrists' experience in using telepsychiatry, was tested it was statistically significant even if there were additional variables in the model. That is, experience is strongly correlated with the behavioral intention to use telepsychiatry.

Altogether, the data suggest that although the behavioral intention of psychiatrists in Israel to use telepsychiatry is relatively low, they perceive themselves as being capable of high performance and that using telepsychiatry will require a low effort. They are interested in using telepsychiatry voluntarily even when it is not mandatory. Experience in using telepsychiatry had the most significant correlation with their behavioral intention to use it. Additionally, facilitating conditions were perceived as being able to make it easier for them to use telepsychiatry and correlated with their intention to use it. Furthermore, they have a positive attitude toward treating patients by telepsychiatry and perceive the risk in using it to be average. There was a gender difference in the correlation with performance expectancy and social influence, where women's behavioral intention to use telepsychiatry was lower than that of men. The age of the psychiatrists and social influence were not found to be influential variables.

## Discussion

The present study aimed to identify barriers or facilitators associated with the assimilation of telepsychiatry use by psychiatrists in Israel. To this end, we used the UTAUT model to examine the behavioral intention to use telepsychiatry in daily practice, and tested various variables that determine this intention to use telepsychiatry. The study showed that variables such as performance expectancy, effort expectancy, voluntariness and attitude toward telepsychiatry, were positively associated with the intention to use telepsychiatry, whereas a lack of experience and facilitating conditions that assist in the use of telepsychiatry were significant barriers to telepsychiatry assimilation.

In the UTAUT framework, performance expectancy was reported to be a determinant of intention to use a new technology (24). In line with the UTAUT model, we found that both effort expectancy and performance expectancy were correlated with behavioral intention. Nonetheless, while the UTAUT model determined that experience was merely a moderator of performance expectancy and social influence, we found that experience alone was strongly associated with the intention to use telepsychiatry. Moreover, the UTAUT model found that social influence is correlated with the intention to use, while in our study we observed that social influence was not significantly correlated with intention to use. We note that the UTAUT model determined that intention to use is a good predictor of actual use. It is possible that these differences stem from the fact that 44.7% of our study participants had some amount of experience with telepsychiatry, correlated with intention to use. This resonates with previous findings that with increasing experience, perceived behavioral control becomes a significant direct determinant of use over and above intention (24).

As found in previous studies, psychiatrists generally supported the idea of conducting consultations by videoconferencing, but raised concerns that video consultations may be less effective than FTF treatment (30). Nonetheless, we found that these expressed attitudes were correlated with prior experience, thus acceptance may increase with use. This finding corresponds with previous reports claiming that those who received training and found it easy and useful to use, were more likely to use telepsychiatry, and that it became easier with use (21, 31, 32). Coinciding with a study by Interian et al. (33), we also found that there were three distinct levels of experience that posed different challenges to the service providers. Those with no direct experience were more likely to be concerned about the fit between videoconferencing modality and mental health practice. Those with limited experience faced initial adoption of services and exposure to logistical barriers, and the most experienced providers reported more instances of patient satisfaction and being able to provide care to patients who otherwise had difficulty accessing care. Inexperience with telepsychiatry can be found in other developed countries, such as the USA, where a study that reviewed 183 residency programs found that only 21 offered any training or experience in telepsychiatry (34).

In the UTAUT model, facilitating conditions were significant predictors of the actual use of technology, but not a significant determinant of the intention to use the system (24). Nonetheless, in our study there was a strong correlation between the degree to which psychiatrists believed that the organization and technical infrastructure support their use of telepsychiatry and their intention to use it. We found that facilitating conditions generate a stronger sense of positivity and success during the initial use of telepsychiatry, associated with higher intention to use telepsychiatry. Unfortunately, most of the public mental health facilities in Israel do not have a strong support system or well-developed infrastructure for the use of telepsychiatry, and it is probable that the responsibility for learning the system will lie largely with the users.

Unlike the UTAUT model, where social influence was found to be a significant predictor for the intention to use technology (24), our study showed that social influence was not associated with the intention to use telepsychiatry. This discrepancy can be explained by the fact that in the two decades that passed since the original UTAUT study, the use of technology has become very widespread and therefore may no longer be a significant barrier to the assimilation of tools such as telepsychiatry. Another possible explanation is that our sample constituted a highly educated population that is used to working with technology and computer systems in everyday work.

The UTAUT model reported a significant correlation of age and gender with the intention to use (24), but we did not observe any of these correlations in our study. However, a more detailed examination of age and gender showed that gender was significantly correlated with performance expectancy and social influence, but not with the other variables, while age was not significantly correlated with any variable. It is plausible that in Israel, even older providers are proficient in implementing communication technology.

Our study encompassed additional variables that were not included in the UTAUT model, such as perceived risk. Unlike studies that reported perceived risk as a considerable factor associated with the intention to use health information technology (26), we did not find perceived risk to be significantly correlated with the intention to use telepsychiatry. In addition, the attitude toward telepsychiatry, which is not addressed in the UTAUT model, was found to be largely positive among Israeli psychiatrists.

To conclude, the present study revealed an intriguing result. Despite multiple factors that favor the use of telepsychiatry (high performance expectancy, low effort expectancy, low perceived risk, a largely positive attitude, high voluntariness, and high expectancy for facilitating conditions) the intention to use was rather low. This awkward result might be explained by the low experience element, which probably plays a pivotal role. Experience can mitigate some of the providers' negative aspects, and contribute to more positive overall attitudes toward an innovation (21). Therefore, we recommend creation of a positive experience for psychiatrists who are beginning to use telepsychiatry, so that the chance of using it in the future will increase. This can be addressed by education and training, which should begin at medical school, and continue with guidance during subsequent use together with continuous, up-to-date support for new developments in the field (21, 33). The opportunity to learn and practice telepsychiatry can encourage providers to be more inclined to use it when appropriate (31). Furthermore, for the provider to experience success in experimenting with a new system, a broader organizational culture is required to provide policy support, including training and administration (35). Hence, the connection between the facilitating conditions and their effects on the continued use of telepsychiatry should be acknowledged, and a training program that provides knowledge about the various aspects of telepsychiatry should be developed.

Beyond learning, training and practice, facilitating conditions can also be directed in various ways to facilitate the daily use of telepsychiatry. For example, support staff could handle ordering and scheduling consultations at both ends for assisting the patient remotely when needed, as well as assisting the service providers [Rolf (36)]. Facilitating conditions can also be a platform that enables easy scheduling and tracking of patients and providers, exchange of clinical data, and data collection and analysis. An example of such is the successful establishment of a web portal, the North Carolina Statewide Telepsychiatry Program (NC-STeP), which facilitates the use of telepsychiatry by patients and providers. It enables navigating and sharing the electronic health records system, and providers can use this platform for scheduling, billing, tracking and reporting encounters, and providing information to health care systems (37).

### Limitations and Future Research

This study has a number of limitations: first, although the sample size is small, it comprises 10% of the active psychiatrists in Israel. Moreover, the demographic and profession-related data indicate an inclusive sample that reflects the full spectrum of Israeli psychiatrists. According to the Ministry of Health (38), female psychiatrists comprised 49% of all psychiatrists in Israel in 2019, similar to the current sample (48.7%). However, only 15% of all psychiatrists were 44 years old or younger, in contrast to the current study, in which 54% of the psychiatrists were 47 years old or younger. It seems that the sample of psychiatrists who participated in the study is slightly younger than the general population of psychiatrists in Israel. Second, there is a possibility that the participants who answered the questionnaire were already interested in telepsychiatry, and therefore their attitude was more positive. Third, self-reporting data are subjective and any given answer may be influenced by self-interest, a lack of knowledge about telepsychiatry or a lack of experience in using telepsychiatry (55.3% psychiatrists without experience). Fourth, the sample used in the study is a convenience sample; therefore, it does not represent all psychiatrists in Israel. Fifth, due to the small *N* we could not test the full UTAUT model and we recommend a future study with a larger number of participants. Finally, the study took place just before the outbreak of the COVID-19 pandemic in Israel and the questionnaires were completed before or at the very beginning of the COVID-19 era. The pandemic resulted in rapid assimilation of telemedicine and telepsychiatry in the world including in Israel (39–41), with higher odds of attending and completing treatment than FTF during this period (42, 43). Mental health providers reported satisfaction with electronic health services (44), patients received telepsychiatry well as a solution for emergency situations when FTF meetings were not possible (45), and in a US study most patients said they would consider it in the future (46). In light of the COVID-19 crisis, the attitudes of the study participants may have changed. While this study reflects the pre-COVID era, when use of telemedicine was less frequent in Israel, it provides an excellent baseline for further studying telepsychiatry. Further research on the post COVID-19 era can, for example, teach us about the actual use of telepsychiatry with respect to the intention to use it, and about the ways in which an emergency is associated with overcoming barriers to the assimilation of new technologies in mental health.

## Data Availability Statement

The raw data supporting the conclusions of this article will be made available by the authors upon request, without undue reservation.

## Ethics Statement

The studies involving human participants were reviewed and approved by University of Haifa Faculty of Health and Social Welfare Ethics Committee. The patients/participants provided their written informed consent to participate in this study.

## Author Contributions

HK and MN conceived the study, designed the experimental approach, supervised the work, and obtained the funding. HK, MN, and MS designed, edited the questionnaires, and wrote the manuscript. HK and MS performed the statistical analyses, disseminated the questionnaires, and approached the psychiatrists. MS collected the completed questionnaires, registered, and gathered the data. All authors contributed to the article and approved the submitted version.

## Funding

This work was funded by the Israel National Institute for Health Policy Research (Grant No. 2018/11/r).

## Conflict of Interest

The authors declare that the research was conducted in the absence of any commercial or financial relationships that could be construed as a potential conflict of interest.

## Publisher's Note

All claims expressed in this article are solely those of the authors and do not necessarily represent those of their affiliated organizations, or those of the publisher, the editors and the reviewers. Any product that may be evaluated in this article, or claim that may be made by its manufacturer, is not guaranteed or endorsed by the publisher.
