# Supplementary material for: The Attitudes and Perceptions of Israeli Psychiatrists Toward Telepsychiatry and Their Behavioral Intention to Use Telepsychiatry
Source: Front Psychiatry. 2022 Mar 21;13:829965. doi: 10.3389/fpsyt.2022.829965 (PMC8977602; doi:10.3389/fpsyt.2022.829965)
Supplement: Supplementary file 1 [file Data_Sheet_1.pdf]

Supplementary Information

Table-1

Independent variable	Definition
Performance expectancy (PE)	The degree to which an individual believes that using the system will help him or her attain gains in job performance
Effort expectancy (EE)	The degree of ease associated with the use of the system
Social influence (SI)	The degree to which an individual perceives that important others believe he or she should use the new system
Facilitating conditions (FC)	The degree to which an individual believes that an organizational and technical infrastructure exists to support the use of the system

The study questionnaires

The UTAUT questionnaire (Venkatesh et al., 2003)

PE1	I would find the system useful in my job
PE2	Using the system will enable me to accomplish tasks more quickly
PE3	Using the system will increase my productivity.
PE4	If I use the system, I will increase my chances of getting a raise.
EE1	My interaction with the system would be clear and understandable
EE2	It would be easy for me to become skillful at using the system.
EE3	I would find the system easy to use
EE4	Learning to operate the system will be easy for me
SI1	People who influence my behavior will think that I should use the system
SI2	People who are important to me will think that I should use the system
SI3	The senior management of this organization will be helpful in the use of the system.
SI4	In general, the organization will support the use of the system.
FC1	I will have the resources necessary to use the system.
FC2	I will have the knowledge necessary to use the system
FC3	The system will be compatible with other systems I use.
FC4	A specific person (or group) is available for assistance with system difficulties.
BI1	I intend to use the system in the next 6 months
BI2	I predict I would use the system in the next 6 months
BI3	I plan to use the system in the next 6 months

The 8 items of Attitude toward Telemedicine in Psychiatry and Psychotherapy (ATiPP) questionnaire (Tonn et al., 2017)

Number	Physicians
1	Generally, telepsychiatry is a good addition to the medical services.
2	For psychiatric or psychotherapeutic issues or mental illness, patient information via Internet or telepsychiatry is very helpful.
3	An effective treatment of the patients with mental illness via Internet or telepsychiatry is possible.
4	The bridging of the waiting time for an appointment in psychiatry/psychotherapy by using the Internet services or telepsychiatry is a sensible option.
5	Aftercare and stabilization after a presence therapy by a psychiatrist or psychotherapist through contact via the Internet or email or telephone are realizable.
6	I would absolutely recommend my patients with psychiatric or psychotherapeutic treatment needs a Web-based intervention or telemedical support, if such were to be offered for the clinical picture.
7	In addition to a face-to-face therapy, an accompanying psychoeducational or psychosocial or additional intervention via the Internet is sensible.
8	An online therapy through the Internet services or telepsychiatry for mental illness can only work effectively with live contact with a therapist through video calling and email or chat.

Experience - How experienced are you in using telepsychiatry?

Perceived voluntariness of use - Would you be interested in using telepsychiatry of your own free will?

Perceived risk - (Egea & González, 2011)

The use of telepsychiatry in my work may pose a risk to the patient's treatment.

The use of telepsychiatry in my work may pose a risk to the patient's diagnosis.

References

Manuel Ortega Egea, J., & Victoria Román González, M. (2011). Explaining physiciansâ€™ acceptance of EHCR systems: An extension of TAM with trust and risk factors. *Computers in Human Behavior*, *27*, 319–332. https://doi.org/10.1016/j.chb.2010.08.010

Tonn, P., Reuter, S. C., Kuchler, I., Reinke, B., Hinkelmann, L., Stöckigt, S., Siemoneit, H., & Schulze, N. (2017). Development of a Questionnaire to Measure the Attitudes of Laypeople, Physicians, and Psychotherapists Toward Telemedicine in Mental Health. *JMIR Mental Health*, *4*(4), e39. https://doi.org/10.2196/mental.6802

Venkatesh, V., Morris, M. G., Davis, G. B., & Davis, F. D. . (2003). USER ACCEPTANCE OF INFORMATION TECHNOLOGY: TOWARD A UNIFIED VIEW^. *Innovation: Management, Policy and Practice*, 425–478. https://doi.org/10.5172/impp.2012.14.2.231
